# Defending scientific integrity through collaboration: the European Medicines Agency and *The Lancet Regional Health — Europe*

**DOI:** 10.1016/j.lanepe.2025.101288

**Published:** 2025-04-01

**Authors:** The Lancet Regional Health – Europe

Scientific progress relies on integrity, independence, and global cooperation. However, political interference in science threatens these principles, challenging the foundations of evidence-based medicine and regulatory decision making. The USA now risks losing its standing as a global leader in scientific research as a result of changes implemented by the Trump administration, which have undermined inclusive science and scientific institutions. Now, more than ever, the global scientific community must unite to protect research integrity and drive progress despite adversity.

The Trump administration has weakened crucial scientific research by withdrawing from WHO, cutting federal funding for gender and race studies, equity, diversity, and inclusion initiatives, and climate science, and suspending diversity programmes in science, technology, engineering, and mathematics. Federal health agencies, including the National Institutes of Health and the US Food and Drug Administration (FDA), face funding cuts and political directives that prioritise ideological agendas over evidence-based decision making.

Many drugs entering the global market undergo early-phase testing in the USA, influencing regulatory considerations worldwide. Although regulatory agencies, including the European Medicines Agency (EMA), conduct independent assessments, US trials often form the initial evidence base for new treatments. However, the lack of inclusion, acknowledgment, or reporting of diverse populations—particularly in terms of sex, gender identity, and race—can limit trial applicability, complicating regulatory evaluations, and raising patient safety concerns. Additionally, proposed FDA restructuring and deregulation might alter oversight, potentially accelerating some drug approvals with fewer regulatory requirements while also causing delays in others due to shifting standards and review processes. If FDA standards diverge substantially from those of other agencies, this could challenge regulatory consistency and weaken the evidence base for new treatments.

In response to these global challenges, Europe has the opportunity to strengthen scientific integrity through regulatory and research partnerships. By reaffirming its commitment to inclusive, unbiased, evidence-based research and policy, Europe can lead the way in upholding transparency and independence, and in promoting inclusive research—principles that are crucial to the future of medical science and public health.

In this spirit, we are pleased to announce a landmark collaboration between the EMA and *The Lancet Regional Health—Europe.* Rooted in our shared commitment to unbiased scientific and regulatory excellence, this partnership aims to disseminate accurate, up-to-date medical knowledge. Both institutions are driven by scientific evidence, rejecting any political interference in regulatory decision making processes, ensuring scientific progress, and transparency.

On Jan 26, 2025, the EMA celebrated its 30th anniversary. As the EU's regulatory authority, it safeguards public health by ensuring the safety, efficacy, and quality of medicines, with decisions grounded in independent, robust evidence. Similarly, *The Lancet Regional Health—Europe* advances health care through inclusive, high-quality research. Together, both institutions uphold the importance of unbiased, evidence-based science in shaping medicine and public health.

By uniting our expertise and influence, we aim to strengthen transparency, foster knowledge exchange, and reinforce the leadership of Europe in research integrity and medical innovation. As part of this collaboration, *The Lancet Regional Health—Europe* will publish news pieces from the EMA every 2 months, offering timely updates on newly approved drugs in the EU. Readers who wish to stay informed can sign-up to our journal alerts here.

To highlight the scale of regulatory decisions in Europe, the EMA recommended authorisation for 114 new medicines in 2024, including 46 containing a new active substance, and expanded indications for 90 existing treatments. Our news pieces will provide timely updates on such developments, ensuring readers stay informed about the evolving regulatory landscape.

The first of these news pieces is included in this issue, covering EMA approvals from January and February, 2025. Highlights include Vimkunya (Chikungunya vaccine (recombinant, adsorbed)), the first vaccine against chikungunya virus for adolescents aged 12 years and older; Vyjuvek (beremagene geperpavec), the first topical gene therapy for dystrophic epidermolysis bullosa; and expanded marketing authorisations for Kaftrio (ivacaftor/tezacaftor/elexacaftor) and Kalydeco (ivacaftor), broadening treatment options for patients with cystic fibrosis.

Additionally, the EMA's approval of ivermectin/albendazole for helminth infections, although not intended for use in the EU, this approval supports regulatory capacity worldwide, enabling non-EU regulators with limited resources to rely on EMA's scientific assessment in their decision making.

Beyond news coverage, this collaboration extends to publishing Reviews (subject to successful peer review) on key areas of importance to European health care. For drugs of heightened public interest, we will also feature Comment pieces with the EMA's perspective keeping the scientific and clinical community engaged with crucial regulatory developments.

Calls for scientific unity are growing louder—and they are urgent. The challenges ahead demand collective action, and our collaboration with the EMA exemplifies how institutions can work together to promote transparency and uphold rigorous scientific standards. Europe must lead by maintaining high-quality research and resisting political interference to keep medical science inclusive, independent, and evidence driven.

